# Supplementary material for: Identification of Freezing-Responsive microRNAs and Their Targets in Chinese Jujube by Small RNA and Degradome Sequencing
Source: Int J Mol Sci. 2026 Sep 4;27(17):7912. doi: 10.3390/ijms27177912 (PMC13566890; doi:10.3390/ijms27177912)
Supplement: Supplementary file 1 [file ijms-27-07912-s001.zip › Supplementary Material S2/novel_miR_204.pdf]

Provisional ID	: novel_miR_204
Score total	: 0.4
Score for star read(s)	: -1.3
Score for read counts	: 0
Score for mfe	: 0.1
Score for randfold	: 1.6
Score for cons. seed	:
Total read count	: 50
Mature read count	: 50
Loop read count	: 0
Star read count	: 0

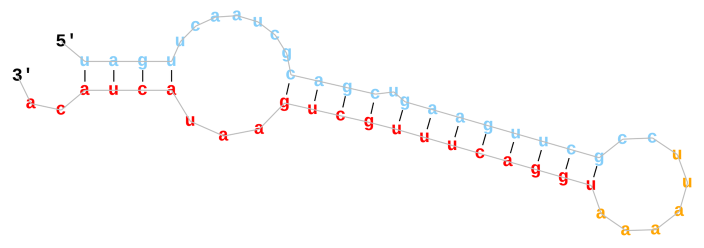

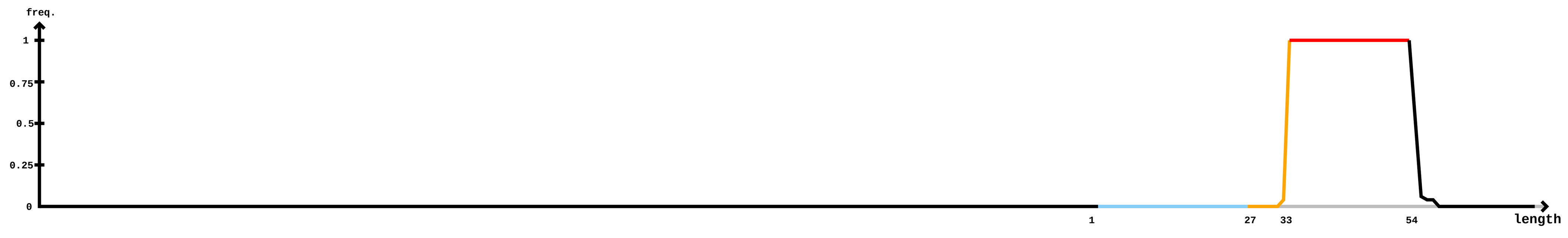

Star Mature

```
5'- agaucauugaguacuuugagguaauuuauccuccugaaauucucuaccauaucaugguuuuucccaaaacaaagaaugcuuugucuuacccuauaccaauucacuugugacaggcaauuccuggggugccaaaaauuauaccgaaguacaacccagcaacauggaugcuugaaguuaguucaaucgcagcugaaguucgccuuaaaauggacuuugcugaauacuacaaaucaucauccuugcaccagua -3'   exp
    ..........((((((((..(((((((..(((((.(.((((.(((..((((...((((..........((((((....))))))..........)))).......))))..))).)))).).)))).)....)))))))..))))))))......((((...(((((..(((...((((.......((((.((((((((........))))))))))))...))))....))).))))))))).......   reads   mm   sample
    ..............................................................................................................................................................................................................auggacuuugcugaauacuacaaauc..................   2   0   JCK-2
    ...............................................................................................................................................................................................................uggacuuugcugaauacuaca......................   25   0   LCK-1
    ...............................................................................................................................................................................................................uggacuuugcugaauacuaca......................   21   0   LCK-2
    ...............................................................................................................................................................................................................uggacuuugcugaauacuaca......................   1   0   LT-3
    ...............................................................................................................................................................................................................uggacuuugcugaauacuacaaa....................   1   0   LT-1
```
